# Resveratrol Increases Sensitivity of Clinical Colistin-Resistant Pseudomonas aeruginosa to Colistin *In Vitro* and *In Vivo*

**DOI:** 10.1128/spectrum.01992-22

**Published:** 2022-12-08

**Authors:** Lingbo Wang, Ying Zhang, Yishuai Lin, Jianming Cao, Chunyan Xu, Liqiong Chen, Yaran Wang, Yao Sun, Xiangkuo Zheng, Yong Liu, Tieli Zhou

**Affiliations:** a Department of Clinical Laboratory, the First Affiliated Hospital of Wenzhou Medical University, Key Laboratory of Clinical Laboratory Diagnosis and Translational Research of Zhejiang Province, Wenzhou, Zhejiang, People’s Republic of China; b Department of Microbiology, Zhejiang Provincial Center for Disease Control and Prevention, Hangzhou, People’s Republic of China; c Department of Medical Lab Science, School of Laboratory Medicine and Life Science, Wenzhou Medical University, Wenzhou, Zhejiang Province, People’s Republic of China; d Wenzhou Institute, University of Chinese Academy of Sciences, Oujiang Laboratory (Zhejiang Lab for Regenerative Medicine, Vision and Brain Health), Wenzhou, Zhejiang, China; Instituto Oswaldo Cruz

**Keywords:** *Pseudomonas aeruginosa*, colistin-resistant, resveratrol, biofilm, synergy effect

## Abstract

Infections caused by colistin-resistant P. aeruginosa strains pose a serious threat to public health. It is therefore urgent to find new strategies to deal with these bacterial infections. We aimed to investigate the efficacy and mechanisms of the colistin/resveratrol combination in eradicating colistin-resistant P. aeruginosa isolates and their biofilms both *in vitro* and *in vivo*. The results revealed that six clinically isolated colistin-resistant P. aeruginosa strains were multidrug resistant (MDR) strains, and resveratrol showed no antimicrobial activity against eight P. aeruginosa strains. Checkerboard assay and time-kill assays indicated that the combination therapy of resveratrol and colistin indicated a remarkable synergistic effect *in vitro*, and biofilm assays and SEM indicated synergistic antibiofilm activity. Furthermore, this combination could efficiently eliminate MDR bacteria in a murine infection model and improve the survival rate of Galleria mellonella. Fluorescence analysis, ALP, and *β*-galactosidase activity test results indicated that the colistin/resveratrol combination increased the membrane permeability of bacteria. In conclusion, our results may provide an efficient alternative pathway against colistin-resistant P. aeruginosa infections.

**IMPORTANCE** P. aeruginosa is a ubiquitous Gram-negative opportunistic pathogen associated with a wide array of life-threatening acute and chronic infections. However, the improper and excessive use of antibiotics has contributed to the increasing emergence of multidrug-resistant (MDR) P. aeruginosa, even colistin-resistant strains, which presents a major challenge to clinical anti-infection treatment. Resveratrol, a naturally occurring polyphenolic antioxidant, can effectively slow down or avoid the occurrence and development of bacterial resistance and is expected to offer a promising strategy to overcome bacterial infections. In this study, colistin/resveratrol combination could synergistically damage the bacterial cell membrane, thereby inducing cell lysis while addressing the emergence of drug resistance. Moreover, this combination therapy may provide an efficient alternative pathway to combat the colistin-resistant P. aeruginosa in clinical practice.

## INTRODUCTION

Pseudomonas aeruginosa (P. aeruginosa) is a ubiquitous Gram-negative opportunistic pathogen associated with several life-threatening acute and chronic infections in humans (1, 2). Because of antibiotic misuse, the increasing emergence of multidrug-resistant (MDR) P. aeruginosa poses a significant challenge for clinical and therapeutics fields (3). Colistin, also known as polymyxin E, efficiently targets lipopolysaccharide (LPS) on outer membrane (OM) and the lipids from bacterial cytoplasmic membrane (4–6). Owing to the massive use of colistin in animal production (7) and clinical practice, colistin-resistant strains have emerging more frequently (8). Therefore, novel approaches that can minimize the toxic side effects of colistin and the emergence of drug resistance in bacteria while increasing the efficacy of colistin are urgently warranted.

In the most infections caused by P. aeruginosa, the presence of biofilm is one of the primary causes of poor efficiency in treatment and reinfections, which lead to high morbidity and mortality rates (9, 10). Biofilms are complex bacterial communities formed on biological and nonliving surfaces and induce antibiotic resistance by hampering the penetration of antibiotics and adapting to diverse various microenvironments (11). As a result, bacteria living in biofilms can be up to 1000 times more recalcitrant to antibacterials than their planktonic counterparts (10). Furthermore, biofilm productions are the major virulence factors associated with the quorum sensing (QS) system, which further aggravate infections caused by drug-resistant bacteria (12). Thus, developing novel anti-biofilm treatment strategies is urgent.

In view of the rapid increase of bacterial resistance, the development of novel antibiotics is both time-consuming and labor-intensive, restricting the therapeutic options for drug-resistant bacterial infections (13). Combining nontraditional antibiotic compounds and antibiotics as a new treatment regimen has thus garnered considerable scientific attention. For instance, resveratrol (3,5,4-trihydroxy-trans-stilbene) is a naturally occurring polyphenolic antioxidant belonging to the stilbene family and is present in several plants, such as peanuts, blueberries and cranberries, and Japanese knotweed (14, 15). Resveratrol has been used as a food preservative and is safe for human consumption (16). Past studies have reported that resveratrol can alter bacterial virulence, reduce membrane integrity, and inhibit biofilm formation (17, 18). Several studies have reported that resveratrol exhibits activity against diverse human pathogens (19). Cannatelli et al. evaluated the synergistic effect of resveratrol and colistin, wherein resveratrol can potentiate colistin activity against colistin-resistant strains (such as Klebsiella pneumoniae, Escherichia coli, Citrobacter braakii, *Stenotrophomonas malthophilia*, Enterobacter *cloaceae*, *and* Acinetobacter baumannii) *in vitro* (20). However, the antibacterial and anti-biofilm activities of the colistin/resveratrol combination against colistin-resistant P. aeruginosa both *in vitro* and *in vivo* have not been investigated previously.

In this study, we investigated the synergistic antimicrobial and antibiofilm effects of the colistin/resveratrol combination against 8 colistin-resistant P. aeruginosa strains *in vitro*, and their potential for *in vivo* synergy was assessed in both mice and Galleria mellonella (G. mellonella) infection models. Moreover, we also preliminarily elucidated the possible mechanism of their synergistic action.

## RESULTS

### Antimicrobial susceptibility.

The MIC of clinical antibiotics for different isolates are presented in Table 1. All 8 colistin-resistant P. aeruginosa strains demonstrated different degrees of drug resistance to the conventional clinical antibiotics. The MICs of colistin for the 8 P. aeruginosa strains were measured at 8 to 64 μg/mL, and 75% (6/8) of these strains exhibited MDR phenotypes. Among the tested clinical antibiotics, these isolates showed the highest resistance of up to 75% (6/8) to imipenem and showed good susceptibility to amikacin and cefepime with resistance rates of 12.5% (1/8) and 12.5% (1/8), respectively. Furthermore, resveratrol displayed no antimicrobial activity against these colistin-resistant P. aeruginosa strains (MIC >512 μg/mL).

**TABLE 1:** MIC value of colistin-resistant clinical isolates against clinical antibiotics and resveratrol^*a*^

Strains	MIC values (μg/mL)										
	COL	ATM	CAZ	FEP	IPM	CIP	LVX	GEN	TOB	AMK	RSV
TL-1671	8^R^	8	4	8	2	0.25	1	2	1.0	8	>512
TL-1736	8^R^	8	32^R^	2	16^R^	1	1	32^R^	8	32	>512
TL-1744	32^R^	32^R^	32^R^	16	16^R^	32^R^	8^R^	>128^R^	32^R^	≤2	>512
TL-2314	8^R^	16	32^R^	16	4	0.5	2	8	2	32	>512
TL-2917	8^R^	32^R^	16	16	16^R^	0.25	2	8	8	4	>512
TL-2967	8^R^	128^R^	16	32^R^	16^R^	8^R^	16^R^	8	8	16	>512
TL-3008	32^R^	4	2	4	16^R^	0.5	1	16^R^	4	32	>512
TL-3086	64^R^	128^R^	16	16	>128^R^	16^R^	8^R^	>128^R^	128^R^	>128^R^	>512

a COL, colistin; ATM, aztreonam; CAZ, ceftazidime; FEP, cefepime; IPM, imipenem; CIP, ciprofloxacin; LVX, levofloxacin; GEN, gentamicin; TOB, tobramycin; AMK, amikacin; RSV, resveratrol; superscript “R” indicates resistance.

### Evaluation of synergy by the checkerboard assay.

The checkerboard assay displayed that the colistin/resveratrol combination exhibited a significant synergistic effect on all 8 colistin-resistant P. aeruginosa strains. As shown in Table 2, the MIC of colistin exhibited a significant (8 to 16-fold) decrease in the presence of resveratrol, and the FICI of the 8 P. aeruginosa ranged from 0.25 to 0.38 (synergy).

**TABLE 2:** Summary of MIC values and FICI for colistin/resveratrol combinations against colistin-resistant Pseudomonas aeruginosa strains^*a*^

Strains	MIC of Monotherapy(μg/mL)		MIC of combination (μg/mL)	FICI	Interpretation
	Colistin	Resveratrol	Colistin/resveratrol		
TL-1671	8	>512	1/128	<0.38	Synergy
TL-1736	8	>512	1/128	<0.38	Synergy
TL-1744	32	>512	2/128	<0.31	Synergy
TL-2314	8	>512	1/128	<0.38	Synergy
TL-2917	8	>512	1/64	<0.25	Synergy
TL-2967	8	>512	1/64	<0.25	Synergy
TL-3008	32	>512	2/128	<0.38	Synergy
TL-3086	64	>512	8/64	<0.25	Synergy
ATCC27853	0.5	>512	0.5/>512	2	Irrelevant

a FICI, fractional inhibitory concentration index.

**FIG 1:**
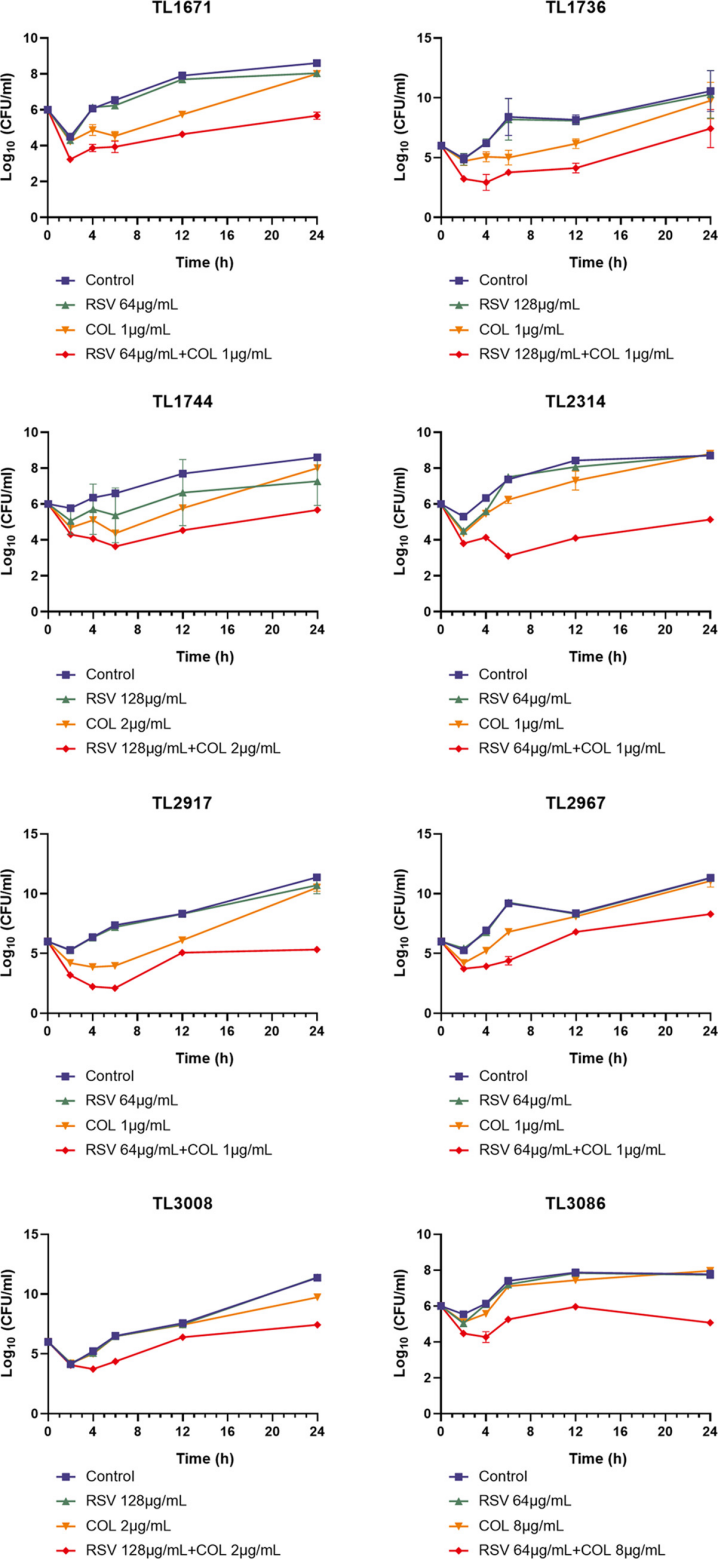
Time-killing curves of COL (colistin) and RSV (resveratrol) either alone or in combination against 8 colistin-resistant P. aeruginosa.

**(i) Synergistic effect of colistin/resveratrol combination by time-kill assay.** We further investigated the *in vitro* synergistic effect of colistin/resveratrol combination against 8 colistin-resistant P. aeruginosa strains by performing the time-kill assay. The drug concentrations used in the time-kill curve were based on the results of the checkerboard assay. As shown in Fig. 1, in the absence of colistin exposure (control group and resveratrol-treated group), all strains grew well. Moreover, in the presence of colistin alone, TL1736, TL1744, and TL2917 exhibited a slight inhibition within the first 6 h. No significant difference was noted among the colistin-treated, control, and resveratrol-treated groups after 24 h of incubation. Moreover, the cell viability of all strains treated with colistin/resveratrol combination dramatically decreased over 2 log_10_ (CFU/mL) within 24 h compared with that in the monotherapy and control groups. Thus, the bactericidal activity of colistin was significantly enhanced when in combination with resveratrol.

### Anti-biofilm activity of the colistin/resveratrol combination.

**FIG 2:**
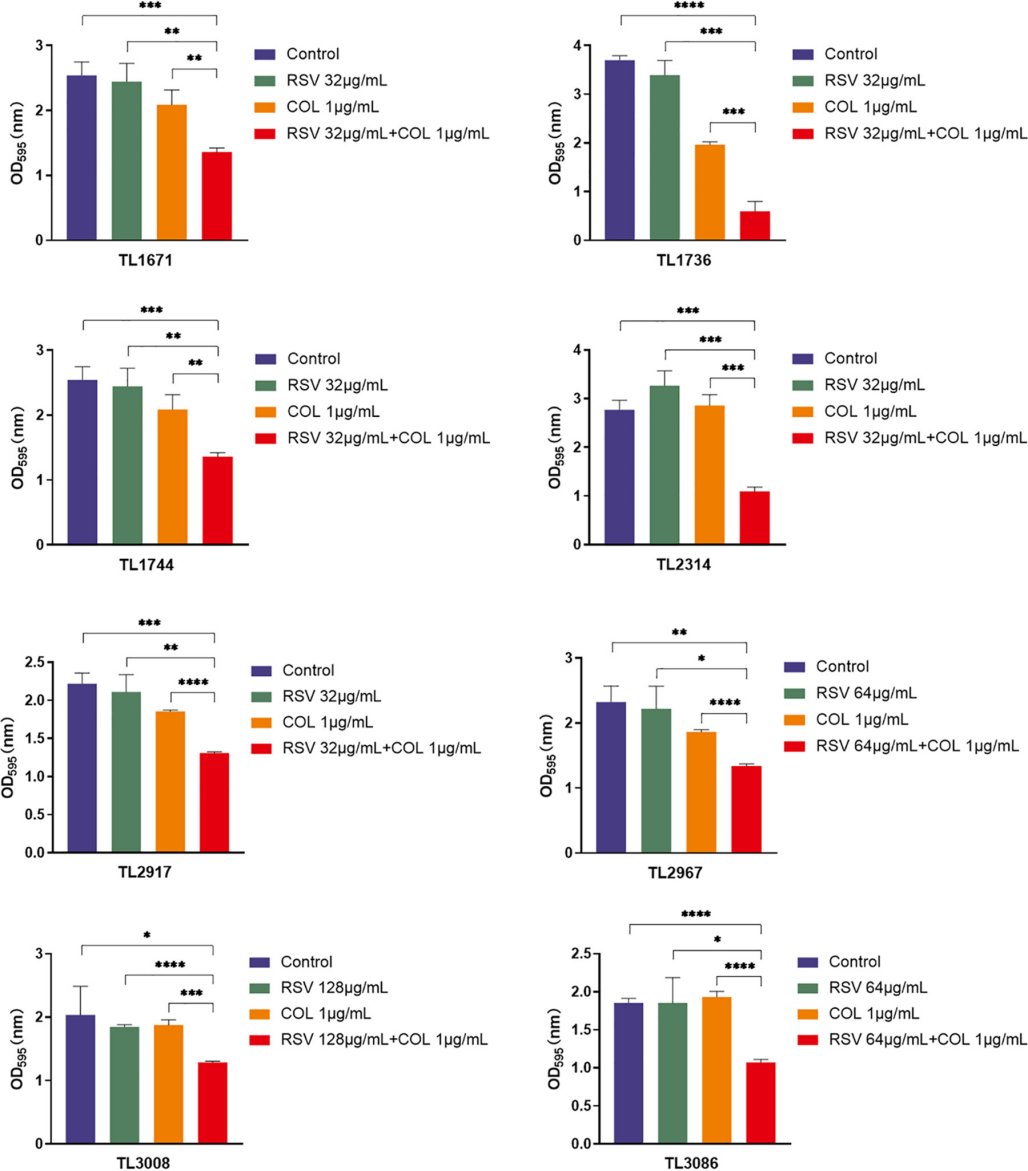
The inhibitory effect of biofilm formation of 8 colistin-resistant P. aeruginosa treated with COL (colistin) and RSV (resveratrol) either in combination or alone at the synergetic concentration based on the results of Checkerboard assays. Different colored bars represent different treatment groups. *P < *0.05 (*), *P < *0.01 (**), and *P < *0.001 (***) analyzed by Student's *t* test.

**FIG 3:**
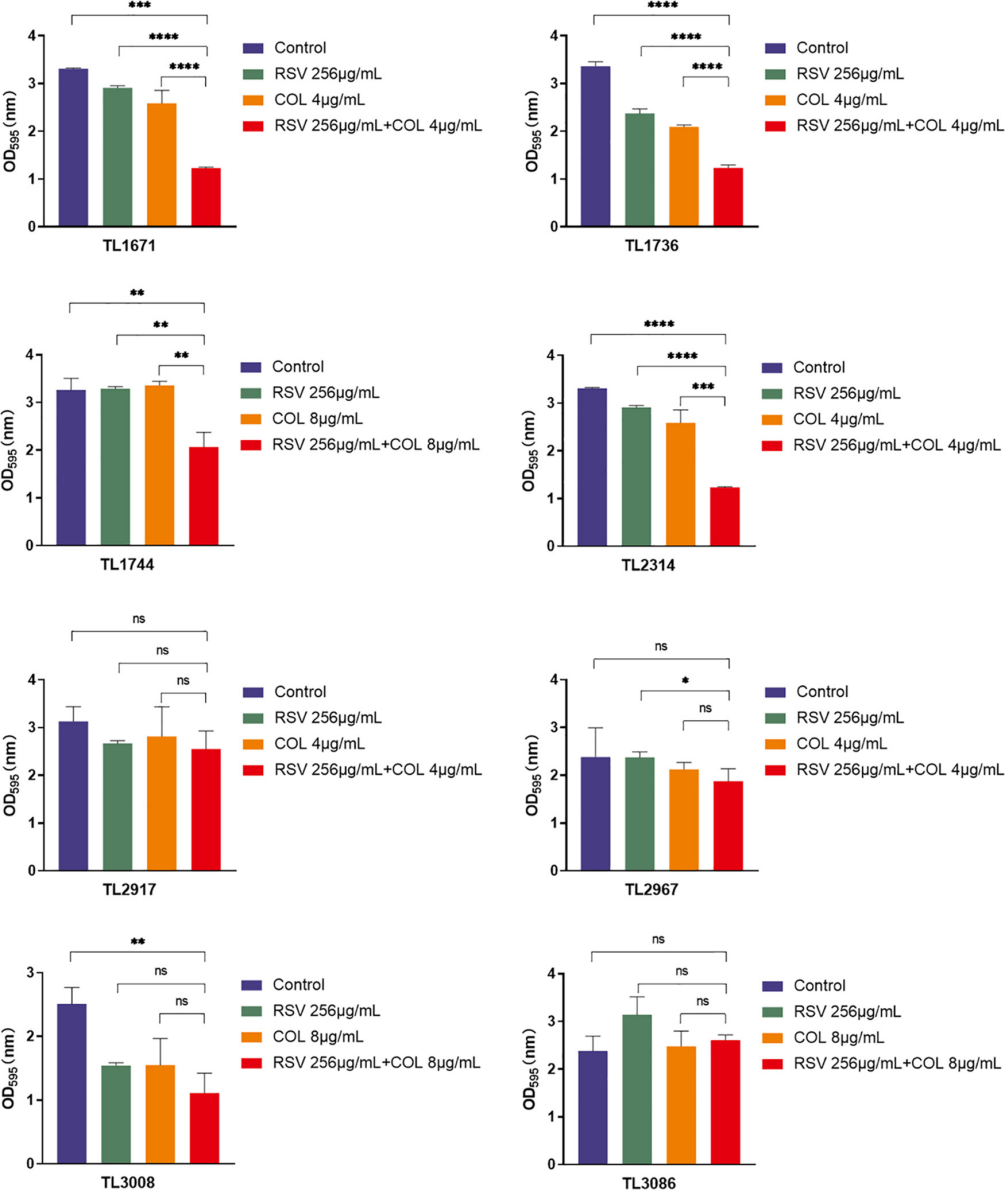
Radiation effect COL (colistin) and RSV (resveratrol) on P. aeruginosa mature biofilm. The selected drug concentration was derived from the Checkerboard method with FICI < 0.5. Different colored bars represent different treatment groups. *P < *0.05 (*), *P < *0.01 (**), and *P < *0.001 (***) analyzed by Student's *t* test.

The effect of resveratrol and colistin on the inhibition of biofilm formation of colistin-resistant P. aeruginosa strains was studied. As shown in Fig. 2, the biofilm formation ability of 8 colistin-resistant P. aeruginosa strains treated with the combination was significantly decreased compared with the control, resveratrol, or colistin groups (*P < *0.05). Notably, as shown in Fig. 3, the combination had a pronounced effect on the eradication of mature biofilms of P. aeruginosa (4/8) compared with that in the control and monotherapy groups (*P < *0.05).

**FIG 4:**
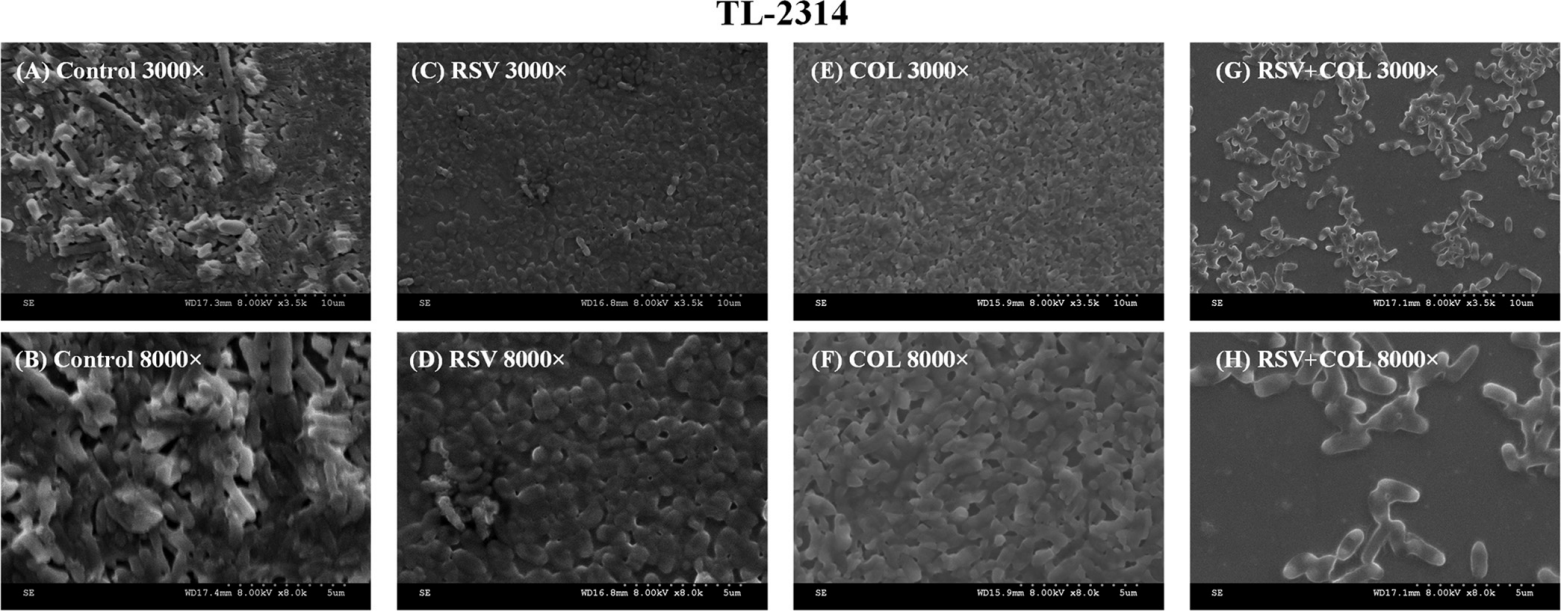
Scanning electron microscopy images of the effects of resveratrol and colistin combined treatment on the bacterial number and biofilm formation of colistin-resistant P. aeruginosa TL-2314. A: LB broth control group, 3,000×; B: LB broth control group, 7,000×; C: Resveratrol monotherapy, 3,000×; D: Resveratrol monotherapy, 7.000×; E: Colistin monotherapy, 3,000×; F: Colistin monotherapy, 7,000×; G: resveratrol/colistin combination, 3,000×; H: resveratrol/colistin combination, 7,000×. RSV (resveratrol) and colistin (COL).

The effect of the combination on the biofilms of the tested P. aeruginosa was further studied by SEM. As shown in Fig. 4, the biofilm of the bacteria (TL2314) treated with resveratrol (32 μg/mL) or colistin (1 μg/mL) was similar to that of the control group, showing complete morphology and a dense arrangement. In contrast, the biofilm treated with the colistin/resveratrol combination exhibited a notable change. The biofilm structure was destroyed, and the number of bacteria was reduced.

### *In vivo* treatment verification.

**FIG 5:**
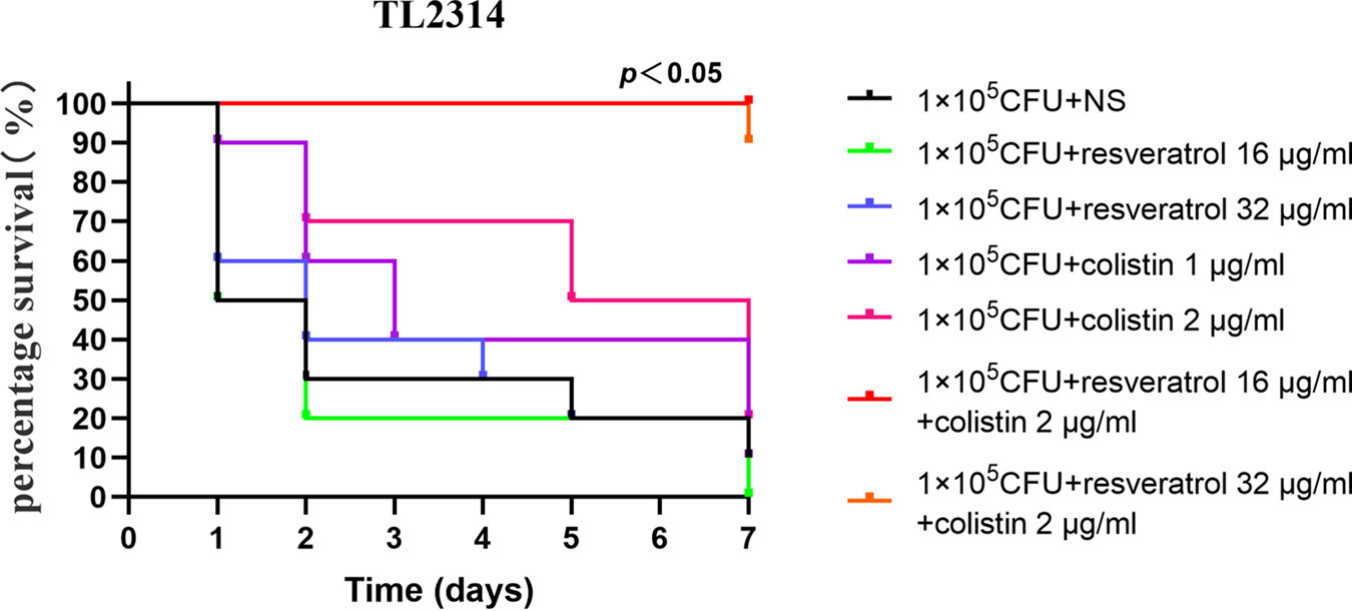
The survival rate of G. mellonella after 7 days of monotherapy or combination therapy using different dosing regimens against colistin-resistant P. aeruginosa TL-2314. Kaplan–Meier analysis and log-rank test performed to analyze the survival rate of G. mellonella larvae. NS: normal saline.

The colistin/resveratrol combination exhibited an apparent synergistic effect *in vitro*, which indicated that need to verify the effects *in vivo*, especially in animal models. First, the results of G. mellonella survival experiments (Fig. 5) indicated that all infected G. mellonella administered with saline showed a low survival rate of 10% within 168 h. The survival rate of G. mellonella administered with resveratrol (16, 32 μg/mL) or colistin (1, 2 μg/mL) was still low (0% to 20%). However, the combination of colistin (2 μg/mL) and resveratrol (16, 32 μg/mL) showed a high survival rate at 90 to 100% within 168 h, it was significantly higher than that of the control group and the monotherapy group therapy (*P < *0.05).

**FIG 6:**
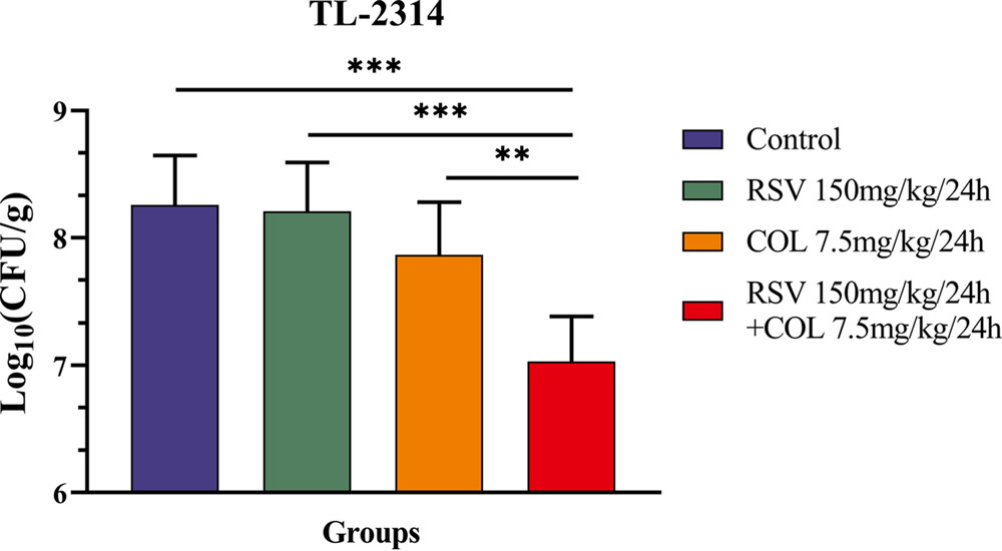
Log_10_ changes in each mouse each thigh muscle (Δlog_10_ CFU/g) after 24 h of monotherapy or combination therapy using different dosing regimens against colistin-resistant P. aeruginosa TL-2314. Each bar represents the data from 3 mice (totaling 6 thighs). Different colored bars represent different treatment groups: blue bar represents control group, green bar represents 150 mg/kg/24h RSV, orange bar represents 7.5 mg/kg/24h COL, red bar represents 150 mg/kg/24h RSV and 7.5 mg/kg/24h COL.

In addition, an *in vivo* murine infection model was developed to further evaluate the efficacy of colistin and resveratrol, either alone or in combination. As shown in Fig. 6, resveratrol at 150 mg/kg and colistin at 7.5 mg/kg slightly inhibited the growth of P. aeruginosa TL2314 within 24 h of treatment. Moreover, the combination exhibited significantly higher efficacies than monotherapy (*P < *0.05). These results indicated that the combination of colistin and resveratrol exhibited a significantly synergistic antibacterial effect in eradicating P. aeruginosa *in vivo*.

### Potential mechanism of the synergy action studies.

**FIG 7:**
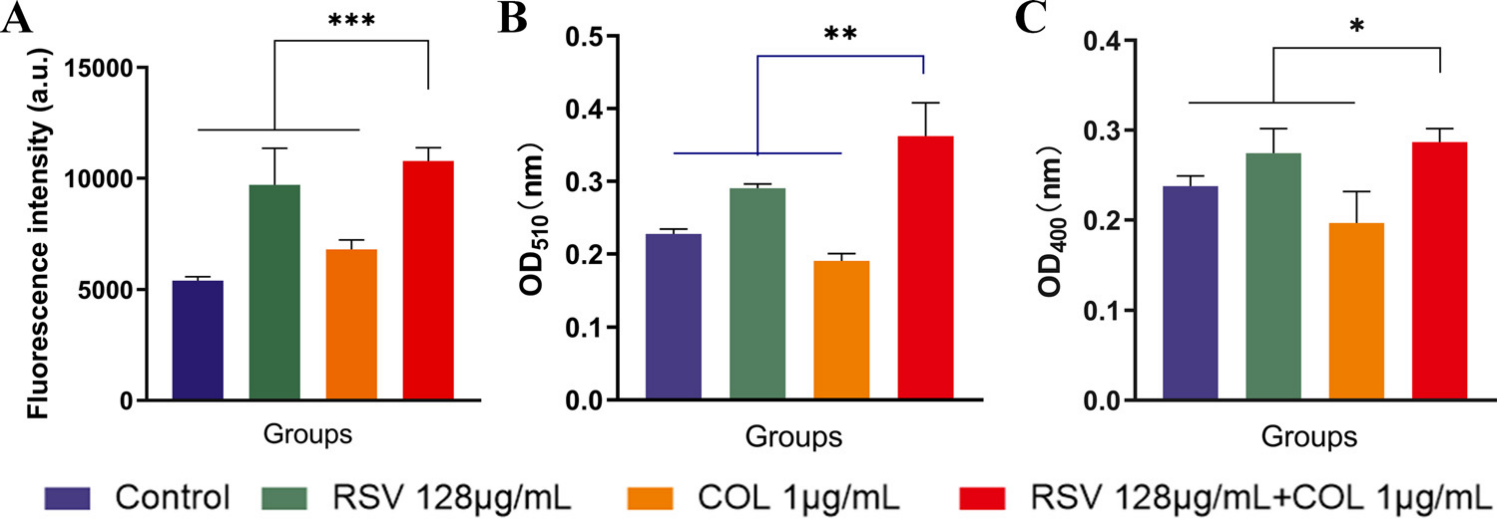
Cell membrane permeability of P. aeruginosa TL2314 detected by PI (A), alkaline phosphatase (B), and β-galactosidase (C) in 128 μg/mL RSV (resveratrol), 1 μg/mL COL (colistin) or 128 μg/mL RSV + 1 μg/mL COL. Different colored bars represent different treatment groups: blue bar represents control group, green bar represents 128 μg/mL RSV, orange bar represents 1 μg/mL COL, red bar represents 128 μg/mL RSV and 1 μg/mL COL.

PI was used to evaluate the cell membrane permeability of P. aeruginosa TL2314 after different treatments. The results (Fig. 7A) indicated that, in the absence of resveratrol exposure (control and colistin-treated groups), the intracellular fluorescence signal was considerably low. The fluorescent signal was enhanced in the colistin/resveratrol combination-treated and resveratrol-treated groups. Despite resveratrol exhibiting no antibacterial activity against colistin-resistant P. aeruginosa, resveratrol modified the membrane structure of the bacteria and increased the membrane permeability, therefore, PI could enter the inside the strain and bind with DNA to emit red fluorescence. The fluorescence signal of the bacteria receiving colistin/resveratrol combination was the strongest, indicating that the cell membrane integrity decreased gradually. These results suggested that resveratrol possibly enhanced the bactericidal effect of colistin through membrane disruption.

Subsequently, the effect of various treatments on extracellular ALPs and β-galactosidases was determined through corresponding assays. As shown in Fig. 7B and C, upon the treatment of resveratrol, the amount of extracellular ALPs and β-galactosidases was notably enhanced in P. aeruginosa, suggesting the damage of the bacterial cell membrane. Furthermore, the colistin/resveratrol combination induced similar or better bacterial cell membrane damage compared with resveratrol monotherapy in all tested isolates.

## DISCUSSION

In recent years, the frequency of MDR Gram-negative bacteria is increasing rapidly, especially because of the misuse of antibiotics, thereby creating significant challenges for clinical and therapeutics researches (21). Moreover, MDR P. aeruginosa is one of the most important bacterial pathogens that cause infections with a high mortality rate (22). Colistin is considered the “last-line” therapy for treating infections caused by MDR Gram-negative pathogens (23). However, the resistance to colistin is constantly increasing among clinical isolates, including P. aeruginosa. Hence, new therapies are urgently warranted to overcome drug resistance of bacteria against colistin. Several studies have reported that the combination of nonantibacterial and antibiotics can be a new treatment strategy for overcoming bacterial drug resistance (24–26).

Resveratrol, a polyphenolic compound present in several plant extracts, exhibited several biological functions, such as anti-inflammatory, anticancer, and antibacterial activities (27). As a QS inhibitor, the activity of resveratrol to inhibit the biofilm and virulence factors of several bacteria has been widely reported (25). Moreover, Uthaibhorn Singkham-in et al. (28) reported that resveratrol could potentiate the chlorhexidine activity against carbapenem-resistant Acinetobacter baumannii by downregulating the AdeB efflux pump. Cannatelli et al. reported that resveratrol could potentiate the colistin activity against several colistin-resistant strains *in vitro*, irrespective of the species and resistance mechanisms (20). However, the emergence of colistin-resistance P. aeruginosa, a pathogen with strong pathogenicity and biofilm formation ability, has garnered considerable attention. The antibacterial and anti-biofilm activities of resveratrol combined with colistin against colistin-resistant P. aeruginosa have not been reported previously. Based on these results, we evaluated the synergistic antibacterial and antibiofilm effects of the colistin/resveratrol combination *in vitro* and *in vivo*. We also preliminarily elucidated the possible mechanism of their synergistic action.

First, we found that the combination therapy of resveratrol and colistin is much more effective than monotherapy of either of the drugs *in vitro*. The antimicrobial susceptibility results indicated that 75% (6/8) of these strains exhibited multiple drug resistance phenotypes and resveratrol did not exhibit antimicrobial activity against 8 colistin-resistant P. aeruginosa strains. However, checkerboard assays showed that the MIC of colistin was reduced by 8- to 16-fold when combined with resveratrol, supporting that the combination therapy could be a promising treatment option for infections caused by colistin-resistant P. aeruginosa. Furthermore, time-kill assays indicated that the combination significantly enhanced the antimicrobial activity compared with that in the control or monotherapy groups.

The increasing emergence and prevalence of MDR P. aeruginosa and the difficulties in eradicating wound biofilms have increased the urgency to investigate effective strategies for overcoming these infections (9). In this study, we investigated the synergistic anti-biofilm activity of resveratrol combined with colistin against colistin-resistant P. aeruginosa. Our results suggested that the colistin/resveratrol combination exhibited a better inhibitory effect on the formation of biofilms. Moreover, this combination could also eradicate preformed mature biofilms of most colistin-resistant P. aeruginosa strains. Moreover, SEM exhibited that the bacterial biofilms were primarily destroyed by the colistin/resveratrol combination (Fig. 4). Since QS regulates the formation of biofilm (12), the improved anti-biofilm activity of the colistin/resveratrol combination may be attributed to the resveratrol's role as a QS inhibitor. In other word, our results are encouraging, and they suggest that the colistin/resveratrol combination can be used to inhibit biofilm formation at an early stage and eradicate biofilms at the later stage.

Considering the excellent synergism of colistin/resveratrol combination *in vitro*, we further developed a murine infection model and G. mellonella infection model to evaluate the antimicrobial efficacy of the combination therapy *in vivo*. Our study results supported that resveratrol/colistin combination could significantly inhibit the growth of strains in the murine infection model. In addition, the survival rate of G. mellonella administered with monotherapy was significantly lower than that in the combination therapy. In other words, the combination of resveratrol and colistin could significantly reduce the amount of colistin, thereby reducing the side effects caused by colistin, indicating that resveratrol has a certain application value in the future clinical treatment. Past studies have reported the effects of resveratrol on chemoprevention, including anti-inflammatory and cytoprotective activities (29). However, its bioavailability is relatively low owing to its rapid metabolization and elimination (30). Its clinical applications are often hindered by the physicochemical limitations such as poor solubility and stability. Therefore, various nanoformulations have been widely investigated to overcome its physicochemical hindrances (31, 32). Huang et al. evaluated the potential of liposomes as a codelivery system for resveratrol. The coencapsulation of pure resveratrol and its cyclodextrin complex in liposomal formulations is a plausible option to enhance the delivery efficiency of the hydrophobic chemotherapeutic agents (33, 34). Moreover, molecular modifications are helpful for improving the bioavailability of resveratrol for future clinical treatment of this combination *in vivo*.

Several studies have reported that nonantibacterial agents and antibiotics combinations exhibit an excellent synergistic effect on MDR bacteria (24, 25). However, the mechanism underlying the synergistic action of such combinations remain unclear. The rapid penetration of antibiotics is the main factor affecting bactericidal activity. Hence, we further explored the mechanism underlying the synergistic action via PI staining and ALP and *β*-galactosidase activity test. Our results indicated that resveratrol possibly enhanced the bactericidal effect of colistin via the membrane disruptive mechanism.

The combination of resveratrol and colistin exhibited an excellent synergistic effect both *in vitro* and *in vivo*. However, this study has some limitations. The synergy between colistin and resveratrol *in vivo* warrants further experimental evaluation and confirmation using different animal models of infection and different dosage combinations. Moreover, its potential clinical practice warrants further investigation.

### Conclusion.

This study is the first to report the antimicrobial and antibiofilm properties of the colistin/resveratrol combination against colistin-resistant P. aeruginosa. Resveratrol potentiated colistin activity against colistin-resistant P. aeruginosa *in vitro* and enhanced the colistin's efficacy *in vivo*. The present study's findings provide insights to the suitability of this colistin/resveratrol combination therapy as an alternative therapy for infections caused by colistin-resistant bacteria.

## MATERIALS AND METHODS

### Bacterial isolates and growth conditions.

A total of eight nonrepeated clinically isolated strains of colistin-resistant P. aeruginosa were isolated from the First Affiliated Hospital of Wenzhou Medical University in China from 2015 to 2017. These eight colistin-resistant P. aeruginosa strains were screened from 736 P. aeruginosa strains using the broth microdilution method. All species identification was performed using the Matrix-Assisted Laser Desorption/Ionization Time-Of-Flight Mass Spectrometry (MALDI-TOF MS, bioMérieux, France). All strains were frozen in Luria-Bertani (LB) broth medium (Oxoid, Britain) supplemented with 30% glycerin at −80°C until further use. P. aeruginosa ATCC 27853 (Clinical and Laboratory Standards Institute CLSI) served as the quality control strain. All the investigation protocols used in this study were approved by the Ethics Committee of the First Affiliated Hospital of the Wenzhou Medical University.

### Antibiotics and solvents.

Resveratrol was purchased from Shanghai Yuanye Biotechnology Co., Ltd. (Shanghai, China) and dissolved in dimethyl sulfoxide (DMSO, ≤2.5% [vol/vol]) (Sigma-Aldrich, Saint Louis, USA). All antibiotics used in this study, including colistin, aztreonam, ceftazidime, cefepime, imipenem, ciprofloxacin, levofloxacin, gentamicin, tobramycin, and amikacin, were purchased from Wenzhou Kangtai Biological Technology Co., Ltd. (Zhejiang, China). Solvents and diluents used to prepare the antibiotic solution complied with the Clinical and Laboratory Standards Institute (CLSI 2020).

### Determination of the antimicrobial resistance profiles.

The MIC of antibiotics and resveratrol against the 8 colistin-resistant P. aeruginosa was determined by using the broth microdilution in cation-adjusted Mueller–Hinton Broth (CAMHB) (Thermo Fisher Scientific, America). Briefly, 96-well microtiter plates containing a series of diluted antibiotics and resveratrol were prepared. Then, 100 μL of the bacterial suspension (1.5× 10^8^ CFU (CFU/mL) was added to 96-well microplates and incubated at 37°C for 16 to 18 h. The breakpoint score was interpreted in accordance with the Clinical and Laboratory Standards Institute (CLSI, 2020). Each MIC test against all isolates was performed in triplicate.

### Checkerboard assays.

The checkerboard method was used to evaluate the *in vitro* synergy between resveratrol and colistin, as previously described albeit with some modifications (35). Briefly, the two drugs were diluted with CAMHB into a series of concentrations based on the MIC for each tested strain. A single overnight-grown bacterial colony was diluted to 0.5 McFarland with sterile saline and subsequently diluted by 1:100 in CAMHB. Each well was inoculated with a final concentration of approximately 7.5 × 10^5^ CFU/mL. The results were observed after incubation of the plates at 37°C for 16 to 20 h. Each test was performed in triplicate.

Synergy was evaluated by calculating the fractional inhibitory concentration index (FICI): FICI = MIC of drug A in combination/MIC of drug A alone + MIC of drug B in combination/MIC of drug B alone. (FICI ≤ 0.5 was considered synergy, 0.5 < FICI ≤ 1, additive effect; 1 < FICI ≤ 2, irrelevant effect; FICI > 2, antagonistic effect) (Qu et al., 2019).

### Time-kill assay.

The time-kill assay was performed to evaluate the synergistic effect between resveratrol and colistin in accordance with the results of the checkerboard assay, as described previously with some minor modifications. (36) Briefly, the 8 colistin-resistant P. aeruginosa were inoculated into 20 mL of CAMHB containing resveratrol and colistin either alone or in combinations. Tubes containing the LB medium alone served as the negative control. The bacterial suspensions were incubated at 37°C with moderate shaking for 2, 4, 6, 12, and 24 h. According to the growth rate of bacteria, appropriate dilution concentration was prepared. At the corresponding time point, 100 μL of the bacterial suspension was spotted on the MH agar plates, and the CFU was counted after incubating the plate overnight at 37°C. A decrease by ≥3 log_10_ for CFU/mL over 24 h was defined as bactericidal activity. A decrease by ≥2 log_10_ for CFU/mL was defined as the synergistic activity (37). All studies were conducted in duplicate.

### Biofilm formation inhibition assay.

Biofilm formation assays were performed in 96-well polystyrene microtiter plates, as previously described (12) with minor modifications. The single colony on the blood plate was shaken overnight in 3 mL of fresh LB broth medium at 37°C. Then, the culture was adjusted to 0.5 McFarland with sterile normal saline and further diluted by 1:100 in LB broth and dispensed in a 96-well microtiter plate with 32, 64, or 128 μg/mL resveratrol and 1 μg/mL colistin either alone or in combinations. The 96-well plates were incubated at 37°C for 24 h. Then the cell suspension was removed, and the plates were washed twice with 1× phosphate-buffered saline (PBS) (Sigma–Aldrich, Milan, Italy) and inverted to dry at the room temperature. Next, 200 μL of 1% crystal violet (CV) solution (Beijing Solarbio Biotechnology Co., Ltd., China) was added to the wells for 15 min. After staining, CV was removed, and the wells were washed thrice with 1× PBS. The plate was dried naturally at room temperature, and the bound CV was solubilized by adding 200 μL of ethanol–acetone (96:5 vol/vol) solution. The absorbance of CV was read at 595 nm on a microplate reader (Multiskan FC). All tests were performed in triplicate.

### Mature biofilm eradication assays.

We further detected the eradication effect between resveratrol and colistin on mature biofilms as suggested elsewhere (38) with minor modifications. Briefly, the eight P. aeruginosa overnight culture in 3 mL of fresh LB broth medium was adjusted to 0.5 McFarland with sterile normal saline, followed by further 1:100 dilution in the LB medium and dispensing in a 96-well microtiter plate. After 24 h of static incubation at 37°C (the formation of mature biofilms), the supernatant was discarded and the plates were washed thrice with 0.9% saline to remove the unattached cells. Next, fresh LB broth containing 256 μg/mL of resveratrol and 4 or 8 μg/mL of colistin either alone or in combination were added to each well (200 μL/well). The LB broth medium without antimicrobials served as control, and the 96-well plates were incubated for 24 h at 37°C. Subsequent CV staining and treatment were performed as described earlier. Each assay was performed in triplicate.

### Scanning electron microscopy (SEM).

To evaluate the morphological changes in the bacterial biofilm after different treatments, TL2314 strain was randomly selected by SEM. The overnight culture in the LB medium incubated at 37°C was adjusted to 0.5 McFarland with sterile normal saline and sterile coverslips were placed in each well of a 6-well plate to which 100 μL of diluted culture plus 1900 μL resveratrol (32 μg/mL) and colistin (1 μg/mL) (either alone or in combination) were added. LB broth served as the negative-control group. Biofilms were grown on the coverslips at 37°C for 18 to 24 h. Then, the bacterial culture was removed and washed with 1× PBS. The biofilm samples were fixed with 2.5% glutaraldehyde fixation solution in a fresh 6-well plate and incubated at 4°C for 4 h. The samples were subjected to an ethanol dehydration series of 30, 50, 70, 90, and 100% (vol/vol) ethanol for 10 min. All samples were then dried for 2 h, and all samples were viewed by SEM (S-3000N, Japan) (24).

**(i) *In vivo* evaluation of synergy in G. mellonella infection model.** We detected the efficacy of resveratrol and colistin in G. mellonella infection *in vivo* through a survival assay, as described previously (39) with some modifications. Healthy larvae weighing at least 250 mg and free of any gray markings were selected at random for the experiments. Then, 10 G. mellonella were randomly selected from each group. Overnight cultures of P. aeruginosa TL2314 was washed with PBS and further adjusted with PBS to a concentration of 1 × 10^5^ CFU/mL. The insects were injected with PBS and used as control. We considered an average hemolymph volume of 50 μL and the increase in the volume caused contributed by bacterial and antibiotic injections (each 10 μL). For example, 10 μL of an antibiotic solution with seven times greater concentration was injected into the larvae (40). Then, 10 μL of the bacteria solution was injected into the rear left proleg of G. mellonella by using a microinjector, followed by the test of the drug alone (resveratrol 16. 32 μg/mL ×7; colistin 1. 2 μg/mL × 7) or in combination (resveratrol 16 μg/mL + colistin 1 μg/mL, resveratrol 32 μg/mL +colistin 2 μg/mL, ×7) of 7 MIC within 2 h of infection. G. mellonella were stored at 37°C and recorded their survival rate was recorded after 24, 48, 72, 96, 120, 144 h, and 168 h. Resveratrol was dissolved in 2% DMSO as our past experiments showed that 2% DMSO had no toxic effect on the G. mellonella compared with saline. All experiments were conducted in triplicate. The larvae were considered dead when they repeatedly failed to respond to physical stimuli. Kaplan–Meier analysis and log-rank test were performed to analyze the survival rate of G. mellonella larvae.

**(ii) *In vivo* evaluation of synergy in mice infection model.** To establish a neutropenic mouse thigh infection model, specific-pathogen-free (SPF) female ICR (Institute of Cancer Research) mice aged 5 to 6 weeks old (Charles River, Hangzhou, China) were used for the *in vivo* studies. The mice were maintained as per the National Standards for Laboratory Animals of China (GB 14925–2010). All animal studies were approved by the Zhejiang Association for Science and Technology SYXK (ID: SYXK [Zhejiang] 2018-0017) and conducted in accordance with the Wenzhou Laboratory Animal Welfare and Ethics guidelines.

Briefly, the mice were injected with cyclophosphamide (Yuanye Biotechnology Co., Ltd., Shanghai, China) intraperitoneally for 4 days (150 mg/kg) and 1 day (100 mg/kg) to induce a neutropenia model (neutrophils ≤ 100/mm^3^). P. aeruginosa TL2314 was selected as the experimental strain, and the mice were categorized into 4 groups (3 mice per group). Each posterior thigh muscle of the mice was exponentially injected with 100 μL of the bacterial suspension (1.5 × 10^7^ CFU/mL). At 2-h postbacterial inoculation, the mice were administered with (a) 1×PBS (untreated group), (b) resveratrol (150 mg/kg), (c) colistin (7.5 mg/kg), and (d) colistin/resveratrol combination (7.5 mg/kg colistin + 150 mg/kg resveratrol) via intraperitoneal injection. The mice were euthanized at 24 h after treatment, and the bacterial burden was quantified based on the CFU counts from posterior thigh homogenates.

### Propidium iodide (PI) staining.

We examined the cell membrane permeability by PI as described previously with modifications (41). We examined the cell membrane permeability by PI as described previously with modifications (41). Briefly, exponential-phase P. aeruginosa TL2314 cells were cultured at 200 rpm at 37°C. Then, the bacterial suspension was divided into four groups, each with 3 mL and treated with the test drug alone (resveratrol 128 μg/mL; colistin 1 μg/mL) or in combination for 2 h. After washing with 1 mL of PBS, the supernatant was discarded and 500 μL PI (50 mg/L) was added and incubated at room temperature for 30 min. Subsequently, the fluorescence intensity of each treatment group was detected at the excitation wavelength of 535 nm and an emission wavelength of 615 nm by the Multifunctional Microplate Reader (Bio Tek).

### Alkaline phosphatase (ALP) activity test.

To analyze the mechanisms of resveratrol acting in synergistic action with colistin, we further evaluated the damage of different treatments on the bacterial cell membrane TL-2314 (OD_600_ = 0.5) were inoculated into an LB broth containing resveratrol (128 μg/mL), colistin (1 μg/mL), and resveratrol (128 μg/mL) + colistin (1 μg/mL), followed by incubation in a shaker (180 rpm) at 37°C for 24 h. Then, these suspensions were centrifuged at 10,000 rpm for 10 min. The supernatant was collected and subjected to the alkaline phosphatase (ALP) activity test by using the corresponding kit (Solarbio, Beijing). All experiments were performed in triplicate.

### β-galactosidase activity test.

Similarly, the *β*-galactosidase activity in the supernatant was tested using its corresponding kit (Solarbio). β-galactosidase decomposed *p*-nitrobenzene-*β*-d-galactopyranoside into *p*-nitrophenol, and the activity of *β*-galactosidase was calculated by measuring its absorbance at 400 nm.

### Statistical analysis.

Statistical analyses were evaluated using the GraphPad Prism 8.0.1 software program. Significance was determined by using two-sample *t* test and log-rank test, with *P < *0.05 (noted with*), *P < *0.01 (noted with**), and *P < *0.001 (noted with***) indicating statistical significance.

### Data availability.

All data generated or analyzed during this study are included in this published article.
